# A Treatise on Hygiene, Public and Private

**Published:** 1845-01

**Authors:** 


### Art. V.

*Traité d'Hygiène publique et privée.* Par Michel Lévy, Médecin ordinaire de premier classe, &c. &c. Tome Premier.—*Paris*, 1844.

*A Treatise on Hygiene, public and private.* By M. Lévy, Physician to the Forces, Professor of Hygiene and of Forensic Medicine, &c. &c. Volume the First.—*Paris*, 1844. 8vo, pp. 602.

Hygiene is important in all its aspects, whether we consider it theoretically, as the final cause of the medical sciences, or practically, as applying the latter to the greatest possible utility. Our sense of its value has been sufficiently expressed by our frequent consideration of the science in general, and of its numerous subdivisions. The works of Virey, Du Chatelet, and Motard on public and general hygiene, and of Dunglison and Kilgour on private hygiene, were reviewed in vols. I, V, and XIV of our Journal. The preservation of health, a special division of the subject has been considered by several modern writers, all of whose works we have noticed more or less at length: amongst these may be enumerated Dr. Southwood Smith, Dr. Andrew Combe, Dr. Hodgkin, Mr. Mayo, and Dr. James Johnson. Dietetics and the physiology of digestion, with reference to diet, have also had their due share of our consideration as a special division of private hygiene. In vols. I, V, and XVII will be found reviews of the works of Doctors Paris, A. Combe, Robertson, Pereira, Davidson, and Truman. The hygiene of infancy, (vol. X), of young females (vol. V), of intermarriage (vol. VII), of religion and mental culture (vols. III, IV, XVI), and other minor questions of this kind have not been neglected by us. The influence of every climate and region has been specially elucidated by the elaborate statistics of the army and navy of the empire, which Major Tulloch and Dr. Wilson have reduced from government reports. Ample notices of these unique climateric data will be found in vols. VIII, IX, XI, XV of our Journal. In addition to these we have noticed (vol. XI) similar military statistics of the climate of Senegal, by M. Thevenot, surgeon in the French navy; of Sierra Leone, by Mr. Boyle, colonial surgeon in Sierra Leone; and of the climate of the United States by Dr. Forry (vol. XVI.) The interesting inaugural dissertation of Dr. Thompson (vide vol. VI) belongs also to this head. We discussed at length (vol. XIII) the works of Drs. J. Johnson, Annesley, and other writers on the diseases of India; nor have overlooked the climateric fevers, as is proved by our reviews of the works of Maillot, Kremers, Manni, Evans, Smith, &c. in vols. IV, VIII and XII. Our notice (in vol. XIV) of Dr. Shapter's admirable essay on the 'Climate of the South of Devon' will serve to show that we have not neglected home products. Nor must we omit to notice in our enumeration of works treating of this branch of hygiene the standard and classical treatise of Sir James Clark (vide vol. XII)—a work of special value, as treating of the therapeutical effects of climate, while the preceding are confined almost exclusively to the pathological. Farther, the influence of age and of seasons in disease was noticed in a review of Dr. Fenger's work (in vol. XIV) as well in the treatises on hygiene as in articles indirectly connected with the subject. The influence of manufactures, trades and professions, have received due attention from us; indeed we can refer with perfect satisfaction to our articles on the works of Lombard (vol. I), Fuchs (vol. X) and Ure, Thackeray, Chadwick, &c. (vol. XV.)

Whilst the publications above enumerated refer principally to private hygiene, public hygiene or state medicine has not been forgotten. The population question is discussed in vols. XIV and XVI. The vital statistics of England as exhibited in the reports of the Registrar-General, of Glasgow by Dr. Cowan (vol. XI), the works of Quetelet, and Edmonds, or social physics (vol. III), the laws of contagion and quarantine (vol. XVI), the biological effects of drainage (vol. XI), the state and noxious influence of our grave-yards, as developed by Mr. Walker (vol. X), by the recent parliamentary committee of inquiry, and by Riecke (vol. XV), and lastly, by Mr. Chadwick in his elaborate report on the subject (vol. XVII), have all been duly noticed. We have also diligently discussed the hygiene of prisons (vols. XII and XVII), of foundling hospitals (vol. XIII), and of hospitals for the insane. We refer with perfect confidence to vols. VII and IX for elaborate articles on the last-mentioned branch of public hygiene. The hygiene of the labouring population has not been overlooked; we refer to articles in vols. XI and XV. In addition to all these, medical education and the social position of the profession have at all times had our anxious attention. If we thus review our own labours in this important department of medical science with feelings of pride and satisfaction, we humbly trust, nevertheless, that our readers will think those feelings warranted by the facts, *the things done*. Having thus detailed the bibliography of hygiene, as it is to be found in the British and Foreign Medical Review, we may now turn to our author.

In arranging the plan of his work, after an historical notice and definition of hygiene, Professor Levy adopts the old and well-tried division of agents that influence the health into 1, the circumfusa; 2, ingesta; 3, secreta and excreta; 4, acta. Previously, however, to the discussion of these, he considers the individual differences of man, and the first chapter is occupied with the doctrine of temperaments. We have met with physicians who could not distinguish the temperaments; who, in fact, denied their existence. Yet the doctrine is as old as medicine itself, and under one guise or another has entered into all systems, and guided practice in all ages. Professor Lévy discusses slightly its history, and contends for the real existence of temperaments. It is not possible in truth to define in mathematical terms the material conditions of the sanguine, or of the nervous, or lymphatic temperament; yet they do not the less certainly exist. M. Royer Collard, one of the last writers on the subject, if not the last, has attempted to exhibit their characteristics, and their causes, by means of chemical and microscopical analysis. The attempt seems however premature. Professor Lévy adopts the three temperaments defined by Bégin, namely, the lymphatic, nervous, and sanguine. The states of health and life they represent exercise a direct and almost supreme influence over the organism: it is their common characteristic to modify every part of the body and every function as well in disease as in health. They do not, however, exclude the predominant agency of certain organs on the general system; but these are consecutive on some series of morbid phenomena. If the activity of the hepatic system be morbidly altered, a set of general phenomena may arise sufficiently homogeneous and constant to warrant the belief in a bilious temperament. Writers, on such grounds, have indicated a genital temperament, but Professor Lévy classes these constitutional characteristics amongst the idiosyncrasies.

It is essential, however, to remember, firstly, that a temperament may be combined in the same individual with one idiosyncrasy or several; the lymphatic temperament, for example, with the bilious idiosyncrasy. Secondly, the temperaments may be united with each other, as when the nervous is seen in a person whose blood is rich in fibrine and globules. Professor Lévy remarks, that the stumbling-block in practice and the key to individual peculiarities may alike be found in this complication of temperaments with each other, and with idiosyncrasies, in connexion with the variable elements brought into the economy by age, habits, and hereditariness. The true and practical end of hygiene is to modify the temperaments and idiosyncrasies by the creation of new habits or the neutralization of hurtful agencies. When the plethoric idiosyncrasy arises from repletion, the hygienic rules are obvious; so also when the cerebral idiosyncrasy is developed in literary men.

*The sanguineous temperament.* The general characteristics of this temperament is the ease with which all the organic functions are performed. The respiration is deep and full; the sanguification active, digestion quick and easy, innervation well ordered, the movements free and regular. In this, both the greatest harmony of function, and the finest symmetry of body are to be observed. The mind partakes of the bodily characteristics. Gaiety, vivacity, ready imagination, courage, and inconstancy, and more of petulance than deep feeling are seen in persons of the sanguine temperament. It is usually supposed that the vivid colour of the surface indicates an abundance of blood. This, however, is not quite correct; pale persons have sometimes the *internal* character of the sanguine temperament, namely, energy of hematosis, an unusual development of the deep-seated capillaries, salient and well-developed muscles, &c.

It has been supposed that the size of the heart and lungs in persons of the sanguine temperament corresponds to their functional activity. Professor Lévy states that his observations on the young soldiers under his care do not bear out this idea. The sanguine is the temperament most commonly seen amongst them, yet minute and repeated stethoscopic investigations have not shown that the heart in them is of unusual size. It is predominant from its susceptibility; its sympathetic relations are more extended; it responds quickly to all physical and moral excitants. Hence the blushes, as prompt as the emotion which excites them; hence the instantaneous acceleration of the pulse under the finger of the physician, &c. With respect to the lungs, Professor Lévy thinks that it is impossible not to notice that the thorax is more convex anteriorly, larger and deeper, and predominates over the abdomen, the latter being short and shrunk. Largeness and depth of the chest are not however peculiar to the sanguine temperament, for the tall lympathic Alsatians are remarkably broad-shouldered.

What is the composition of the blood in the sanguine? Professor Lévy infers from Andral's experiments that there is an increase of globules, and that it occurs to the extent of 0·140 from the medium quantity 0·127. Professor Lévy also thinks there is an increase in the solid matter of the blood and a diminution of the saline constituents, or rather in the salts of soda and potass.

*The nervous temperament.* If a number of persons be placed under exactly the same circumstances, whether moral or physical, the phenomena of reaction will be seen to differ more or less in each. These varying shades of difference in the phenomena are due to the varying power of the agent, the nervous system. The individuals in whom it is predominantly active are of medium stature, expressive and lively countenance, of thin fibre, small proportions, pale sallow complexion approching to yellowness. The eye is almost always *vif* and restless, the forehead lofty and the whole head disproportionately large compared with the face. If in action, their motions are abrupt, jerking, and surprisingly energetic when compared with the leanness and softness of their muscles. To the touch, the warmth of the surface communicates a sharp and almost biting sensation instead of that feeling of softness and moisture observed in persons of the sanguine temperament.

Two circumstances are considered as belonging specially to the nervous temperament; a considerable encephalic development and special activity in the genital organs. The sexual propensities of "lean dogs" are proverbial, but we were not aware that popular observation on this point had impregnated the doctrines of professors.

The characteristics of the nervous temperament are, of course, mobility —susceptibility of excitation—affectability. The disproportion between the cause of sensations and their effects is usually very great; a slight excitement inducing often extreme derangement of the nervous system. Yet individuals thus endowed are not so debile as their external appearance indicates. They often bear toil and deprivation better than the sanguine; in highly-depressing circumstances, they shine unexpectedly forth as true heroes.

*The lympathic temperament.* This consists in the predominant vitality of all the organs forming the non-sanguineous fluids, as serum, mucus, lymph, &c. It is diametrically opposed to the sanguineous in this, that the heart and vascular system are at a minimum of development. The tissues which should be most abundantly supplied with red blood, as the muscles, are remarkably pale and flaccid. There are fewer red globules in the blood, and more water. The strictly lymphatic individual has fair hair and complexion, the skin is smooth and soft, and marked by veins; the orifices of mucous membranes but slightly coloured, the teeth generally decayed, or of a pale bluish colour; the cheeks patched with red, the manner hesitating, the movements slow, the hands and feet large. Professor Lévy thinks that this temperament is one exhibiting a degradation of the race, and that its causes exist as well in society as in the atmosphere and in special localities.

*The compound temperaments.* The most common is that made by the union of the lymphatic and sanguine. It is the leading characteristic of entire races, as in the upper Rhine, Alsace, and Normandy, and the departments of the North. The remarks of Professor Lévy on idiosyncrasies are interesting, the term including what we should designate as peculiarities of constitution. Doubtless the more limited use of the term with us as to one of the practical results of idiosyncrasy, namely, the special influence of remedies, is less correct than this. If a number of persons be alike exposed to a current of cold air, the pathological results differ according to the idiosyncrasies of each: one will suffer from colic, a second from cough, a third from rheumatism, &c. Predominance of organs will combine with idiosyncrasy or temperaments, as the hepatic with the sanguineous or nervous. According to these views, age modifies the idiosyncrasies as the organs are developed in succession. As to the detection or diagnosis of idiosyncrasies and the practical application of this knowledge to therapeutics and hygiene, little can be said. We have ourselves observed them most frequently in persons of the nervous or sanguineo-nervous temperament with a scrofulous taint.

Professor Lévy goes on to discuss the hygienic relations of age and sex. Under these heads, a good general summary is given, presenting, however, nothing worthy special notice. In stating the recent views respecting the phenomena of menstruation developed by both English and French physiologists, the names of the former, *more Gallico*, are altogether omitted. After a review of the general facts, Professor Lévy admits the doctrine that there is a mensual movement in man.

*Hereditariness.* This chapter commences with some general facts. The second paragraph contains an account of the wonders that Bakewell, "fermier Anglais" worked amongst cattle. Bakewell made up his mind to breed cattle having the "choice cuts," of a "volume énorme" at the expense of inferior joints, and in fifteen years he was able to exhibit a numerous race of kine in which the head and bones were the smallest possible, the legs short, the chest deep, the haunches so largely developed, that the muscular portions alone formed two thirds of the whole weight of the animal. Bakewell "(fermier Anglais) jugea que les cornes de bœufs étaient inutiles et souvent dangereuses; il créa des espèces complétement dépourvues de cornes." Bakewell (to translate our author) decided that the horns of cattle were useless and often dangerous; so he created a species entirely without horns !! Bakewell created, too, that fine race of horses seen drawing the London trucks. But his greatest triumph was his reform of the woolly tribe. The "moutons de Dishley" remain to this day a living monument of Bakewell's creative powers! Such in sober earnest are the statements which a professor of hygiene by concours gravely prints (say 750 times) as well-authenticated facts. We ought to add, however, that they are quoted from a paper by Royer Collard in the memoirs of the Royal Academy of Medicine for 1843. With this exception the chapter on hereditariness contains nothing not already known or acted upon by British practitioners. Professor Lévy adopts the harmless theory of Lallemand as to the mode in which the hereditary predisposition is transmitted. It appears from some remarks of our author that it is one of the fashions of the French to be treated prophylactically for hereditary diseases, and that large sums are expended in advice and "mercurials, martials, antiscorbutics, bitters," &c.

*The influence of habits* in health and disease. Professor Lévy's chapter on this subject contains very interesting remarks. He defines the effect of habit to be ' a new tendency imparted to the organism by the continuance of the same impressions, or by the frequent repetition of the same acts. He justly argues, that this faculty of acquiring new dispositions and tendencies is the basis of human perfectibility. It is alike the moving force of education and the condition most necessary to an efficacious employment of hygiene. Abuse and excess have been generally connected with habits, and philosophers and hygienists have declaimed against them, as if the power to acquire them was not useful, and indeed one of the conservative powers of nature. It is in fact by this, that living organisms become suited to and flourish in the media in which they live. The more complex an organism, the more need of a flexible power of adaptation to external circumstances. It is by the power of habit or custom (the same thing) that man suits himself to all climates and all circumstances, whether social or moral, and assures to himself that amount of happiness which his illimitable desires will allow. Professor Lévy enters into numerous details illustrative of the beneficial power of habit in resisting injurious agencies, as cold, malaria, &c. He also discusses bad habits in detail, beginning with those implicating the organs of generation, as masturbation, venereal excesses, and pollutions, &c.; then those implicating the organs of plastic life, as neumatosis, habitual vomitings (making no mention, however, of ruminant men), diarrhea, constipation, various hemorrhages, &c. Under this head Professor Lévy also reviews habitual discharges considered as salutary and therapeutic. We do not, however, notice anything new in his facts and arguments; nor does he ever rise above an unexceptionable mediocrity. The details most novel and interesting to us are those on nostalgia, an affection of which he seems to have had considerable experience, and the nature and treatment of which he describes in terms far removed from humble philosophic prose.

*The constitution.* The constitution of an individual has been generally confounded with his temperament. It differs, however, according to Professor Lévy, in this that it includes all the peculiarities of an individual; is in fact the general expression, or formula, of his temperament, idiosyncrasies, age, sex, habits, hereditary dispositions. The degree of physical energy, the greater or less perfection of formation, the sum of the resistance made by the organism to the causes of disease, the proportion of vitality, and consequently the probability of life, are all included in this formula. These elements are discussed at length; then the energy peculiar to each temperament and idiosyncrasy is reviewed under separate heads, and various statistical details given, principally from Woillez (noticed in our Seventh vol.) and Messrs. Andral and Gavarret. The researches of M. Quetelet are freely laid under contributiou in subsequent sections, having reference to age, sex, height, &c. Professor Lévy remarks, that one cannot but notice that a certain height is coincident with a generally healthy constitution, and *vice versâ*. The corps of the French army which comprise the picked men are the artillery, engineers, sappers and miners, &c.; they are selected solely because of their higher stature, the regulation height being six inches (French) or nearly one tenth above that of the infantry of the line. These select corps not only present fewer cases of sickness, and have a less proportion of deaths than the infantry, but Professor Lévy says it is impossible to glance along the lines without being struck with the difference between the two classes of troops. The very tall men, the grenadiers, are, however, much less vigorous than the short thick-set men in the light cavalry, the chasseurs, and voltigeurs.

*Morbid imminence.* In the chapter thus headed Professor Lévy discusses the predisposition to disease peculiar to the temperaments, idiosyncrasies, sexes, different ages, &c. There is little in the chapter not known, we should think, to the generality of our readers. We select the following table, exhibiting the distribution of diseases as they appeared in the military under Professor Lévy's care at Val de Grace, the majority of whom are aged between 21 and 35 years. The whole number of cases treated during the two years 1840-41, was 1877.

| | |
|---|---|
| Diseases seated in the cerebro-spinal system and its coverings | 100 |
| ,, organs contained in the chest | 810 |
| ,, ,, ,, abdomen | 837 |
| Various diseases (articular, rheumatism, cutaneous phlegmasiæ, lumbago, &c.) | 130 |
| Total | 1877 |

817 cases treated during the year 1842 were localized as follows:

| | |
|---|---|
| Cerebro-spinal system and the organs of the senses | 33 |
| Pleura and lungs | 212 |
| Heart and pericardium | 7 |
| Digestive organs | 335 |
| Intermittent fevers | 134 |
| Cutaneous phlegmasiæ, acute and chronic | 43 |
| Divers affections | 53 |
| Total | 817 |

The result is that the head was the seat of disease in 133 cases, the chest in 1029 cases, the abdomen in 1172, intermittents not included. A chapter on the management of convalescents closes the first division of the work.

The second division discusses the action and uses of the modifications of health; the materia of hygiene, commencing with the circumfusa, or the air, earth, and water. The composition of the air, its electrical tension, &c., comprising the general principles of meteorology, are noticed, firstly, alone, and then with reference to their hygienic action on man. Our notice will not permit us even to state the plan of the author in detail. Suffice it to say, that like his literary countrymen in general, he adopts a systematic and comprehensive method, leaving few details unnoticed. Professor Lévy treats of waters in the same orderly manner: first, of their nature, under the principal division of pluvial, oceanic, running, and stagnant; secondly, of their action on the organism. So also in noticing the earth or soil; its temperature, electricity, structure, configuration, and state of surface are noticed, firstly, historically, then physiologically and pathologically.

Another article is devoted to *localities*, detailing the meteorological, hydrological, and geological conditions having a hygienic value. Next follows a notice of climates, their limits, seasons, diseases, and therapeutic value, a special chapter being devoted to the theory and practice of acclimation.

We freely accord Professor Lévy the praise of industrious research; and we may particularly mention that the most important of the facts he has collected on climate and the other subjects mentioned, are derived from the medical statistics of the British army and navy, and from the works of British writers on tropical climates. A series of valuable tables on the distribution of heat over the globe also illustrates this section. The months of December, January, and February constitute the winter of the table, and the other months are in like manner distributed over the seasons, after the method we suggested to the Registrar-General in our last review of his Annual Reports.

A chapter on habitations, and the hygiene of free and confined air, concludes the volume. A second, we presume, will complete the work.

---

